# Supplementary material for: Navigating the New Reality: A Review of the Epidemiological, Clinical, and Microbiological Characteristics of Candida auris, with a Focus on Children
Source: J Fungi (Basel). 2023 Jan 28;9(2):176. doi: 10.3390/jof9020176 (PMC9963988; doi:10.3390/jof9020176)
Supplement: Supplementary file 1 [file jof-09-00176-s001.zip › jof-2154032-supplementary.pdf]

**Table S1**: Supplementary data on the clinical characteristics of children with *C. auris* infection or colonization.

| Continent | Country | # of Cases | Age, Mean | Male Sex | Source | Treatment | Preterm | Underlying Conditions | Mortality | Reference |
|---|---|---|---|---|---|---|---|---|---|---|
| South America | Colombia | 34 | NA | 64% | 100% blood | 47% amphotericin B<br>29% azoles<br>21% caspofungin | 26% | 100% Abx<br>82% CVC<br>82% respiratory support<br>59% malnutrition<br>56% TPN<br>32% corticosteroids<br>15% surgery<br>12% malignancy<br>3% renal disease | 41% | Berrio et al. [31] |
| South America | Colombia | 39 | NA (19%) aged <1 yr) | NA | Blood, urine, respiratory, GI system | NA | NA | NA | NA | Escandon et al. [33] |
| South America | Colombia | 8[1] | 16 d | NA | 50% blood (75% with additional site: sternal surgical incision, peritoneal fluid and 1intravascular thrombus)<br>13% abdominal wall incision<br>13% peritoneal tip catheter<br>25% urine | 13% fluconazole<br>63% caspofungin<br>25% fluconazole and caspofungin | 13% | 100% broad spectrum abx<br>88% cardiac anomalies<br>88% respiratory support<br>75% surgery<br>38% CVC<br>25% NEC | 38% | Alvarado-Socarras et al. [30] |
| South America | Colombia | 12[2] | 34 d (median) | 75% | 100% blood | NA | 50% | 100% CVC<br>100% respiratory support<br>92% TPN<br>75% surgery<br>50% cardiac anomalies<br>33% corticosteroids | 42% | Armstrong et al. [25] |

| | | | | | | | | | | |
|---|---|---|---|---|---|---|---|---|---|---|
| | | | | | | | | 8% hemodialysis | | |
| South America | Venezuela | 13 | <2 m and one aged 14 yr | 46% | 100% blood | 31% fluconazole<br>15% caspofungin<br>8% voriconazole<br>31% caspofungin and voriconazole<br>8% fluconazole and anidulafungin<br>8% fluconazole, voriconazole and caspofungin<br>8% voriconazole, caspofungin and amphotericin B<br>8% voriconazole and caspofungin<br>8% amphotericin B and caspofungin<br>8% voriconazole, caspofungin and anidulafungin | 61% | 100% CVC<br>100% previous abx<br>85% sepsis/septic shock<br>46% abdominal surgery<br>15% HIE<br>8% ventriculitis<br>8% RDS<br>8% cardiac anomalies | 31% | Calvo et al. [32] |
| Asia | Iran | 1 | 14 yr | 0% | Ear | Topical nystatin and oral terbinafine | 0% | None | 0% | Abastabar et al. [22] |
| Asia | Iran | 1 | 2.5 yr | 100% | CSF | Fluconazole, amphotericin B and flucytosine | 0% | Congenital Immune-deficiency (CGD) | NA | Mirhendi et al. [29] |
| Asia | India | 17 | 19 d | 70% | 88% blood (40% with additional site: CSF, eye, urine, wound)<br>6% urine<br>6% urine and respiratory tract | 29% amphotericin B and micafungin<br>24% amphotericin B and voriconazole<br>18% voriconazole<br>12% micafungin and voriconazole<br>12% fluconazole<br>6% fluconazole and voriconazole | 88% | 100% CVC<br>94% TPN<br>82% sepsis and abx<br>82% respiratory support<br>47% surgery (abdominal in 75%)<br>29% RDS<br>12% cardiac anomalies | 41% | Chandramati et al [42] |
| Asia | India | 1 | NA | NA | Blood | NA | NA | NA | NA | Kaur et al. [16] |
| Asia | India | 5 | 9 d | 60% | 100% blood | 40% micafungin<br>60% micafungin and voriconazole | 100% | 100% CVC<br>100% TPN<br>100% RDS<br>100% abx | 80% | Ramya et al. [41] |

| | | | | | | | | | | |
|---|---|---|---|---|---|---|---|---|---|---|
| | | | | | | | | 40% NEC<br>20% bacterial sepsis | | |
| Asia | India | 5 | 2 yr | 20% | 100% blood | 60% amphotericin B<br>20% caspofungin<br>20% no treatment | 40% | 100% broad spectrum abx<br>80% CVC<br>80% immune-deficiency (ALL/neutropenia)<br>20% surgery<br>20% GI anomalies<br>20% cardiac anomalies<br>20% respiratory support<br>20% renal disease | 40% | Chowdhary et al. [79] |
| Asia | India | 3 | <1 m | NA | NA | NA | NA | NA | 67% | Singh et al. [28] |
| Asia | India | 22 | NA<br>(27% <1m) | NA | 100% blood | NA | 18.2% | 24%, 50%[3] surgery<br>44% CVC<br>44% respiratory support | 40.9% | Chakrabarti et al. [31] |
| Asia | Bangladesh | 13 | <1 m | NA | 100% blood | NA | NA | NA | NA | Dutta et al. [35] |
| Asia | Bangladesh | 3 | 10 d | 100% | 100% blood | 100% voriconazole | NA | 100% broad spectrum abx | 67% | Sathi et al. [26] |
| Asia | Pakistan | 1 | NA | 100% | Blood | NA | NA | CVC<br>broad spectrum abx | 0% | Moin et al. [44] |
| Asia | Korea | 3 | NA | NA | Ear | NA | NA | NA | NA | Kim et al. [45] |
| Asia | South Korea | 2 | 1 yr | 50% | 100% blood | Fluconazole and amphotericin B | 0% | 100% TPN<br>100% HIE<br>50% broad spectrum abx<br>50% CVC<br>50% respiratory support | 50% | Lee et al. [34] |

| | | | | | | | | | | |
|---|---|---|---|---|---|---|---|---|---|---|
| | | | | | | | | 50% HLH and chemotherapy<br>50% abdominal surgery | | |
| Asia | Oman | 2 | 1 yr | 100% | 100% blood | NA | NA | 100% broad spectrum abx<br>100% urinary catheter<br>50% neutropenia | NA | Mohsin et al. [21] |
| Europe | Italy | 1 | 1 d | 0% | Axilla, skin, eyes and ears | Colonization | 100% | Extreme prematurity<br>HIE | 100%[4] | Mesini et al. [43] |
| South Africa | Gauteng province | 47 | NA (15% <1 m) | NA | 100% blood | NA | NA | NA | NA | Shuping et al. [27] |
| South Africa | Gauteng province | 15 | NA (93% <1 m) | NA | 100% blood | NA | NA | NA | NA | Chibabhai et al. [33] |

Abx-Antibiotics, AmphoB- amphotericin B, CVC-central venous catheter, NA- not available, GI-gastrointestinal, NEC-necrotizing colitis, HIE-hypoxic ischemic encephalopathy, RDS- respiratory distress syndrome, CSF- cerebrospinal fluid, ALL-acute lymphocytic leukemia, CGD- chronic granulomatous disease, TPN-total parenteral nutrition, HLH-hemophagocytic lymphohistiocytosis.
[1] Four included in [17] [Dr. Rodriguez-Morales, Universidad Tecnológica de Pereira, Colombia, personal communication].
[2] Additional 8 aged 1-18 years without separate clinical data.
[3] In neonates and non-neonates, respectively.
[4] Unrelated to *C. auris* isolation.

# References


16. Alvarado-Socarras, J.L.; Vargas-Soler, J.A.; Franco-Paredes, C.; Villegas-Lamus, K.C.; Rojas-Torres, J.P.; Rodriguez-Morales, A.J. A Cluster of Neonatal Infections Caused by *Candida auris* at a Large Referral Center in Colombia. *J. Pediatr. Infect. Dis. Soc.* **2021**, *10*, 549–555. https://doi.org/10.1093/jpids/piaa152.
17. Escandón, P.; Cáceres, D.H.; Espinosa-Bode, A.; Rivera, S.; Armstrong, P.; Vallabhaneni, S.; Berkow, E.L.; Lockhart, S.R.; Chiller, T.; Jackson, B.R.; et al. Notes from the Field: Surveillance for *Candida auris*–Colombia, September 2016-May 2017. *MMWR Morb. Mortal. Wkly. Rep.* **2018**, *67*, 459–460. https://doi.org/10.15585/mmwr.mm6715a6.
21. Mesini, A.; Saffioti, C.; Mariani, M.; Florio, A.; Medici, C.; Moscatelli, A.; Castagnola, E. First Case of *Candida auris* Colonization in a Preterm, Extremely Low-Birth-Weight Newborn after Vaginal Delivery. *J. Fungi* **2021**, *7*, 649. https://doi.org/10.3390/jof7080649.
22. Mirhendi, H.; Charsizadeh, A.; Aboutalebian, S.; Mohammadpour, M.; Nikmanesh, B.; de Groot, T.; Meis, J.F.; Badali, H. South Asian (Clade I) *Candida auris* meningitis in a paediatric patient in Iran with a review of the literature. *Mycoses* **2022**, *65*, 134–139. https://doi.org/10.1111/myc.13396.
25. Ramya, G.M.; Balakrishnan, U.; Chandrasekaran, A.; Abiramalatha, T.; Amboiram, P.; Sekar, U.; UshaDevi, R. *Candida auris*, an emerging pathogen–Challenge in the survival of microprimies. *Indian J. Med. Microbiol.* **2021**, *39*, 367–369. https://doi.org/10.1016/j.ijmmb.2021.03.025.

26. Rodriguez, J.Y.; Le Pape, P.; Lopez, O.; Esquea, K.; Labiosa, A.L.; Alvarez-Moreno, C. *Candida auris*: A Latent Threat to Critically Ill Patients With Coronavirus Disease 2019. *Clin. Infect. Dis.* **2021**, *73*, e2836–e2837. https://doi.org/10.1093/cid/ciaa1595.
27. Armstrong, P.A.; Rivera, S.M.; Escandon, P.; Caceres, D.H.; Chow, N.; Stuckey, M.J.; Díaz, J.; Gomez, A.; Vélez, N.; Espinosa-Bode, A.; et al. Hospital-Associated Multicenter Outbreak of Emerging Fungus *Candida auris*, Colombia, 2016. *Emerg. Infect. Dis.* **2019**, *25*, 1339–1346. https://doi.org/10.3201/eid2507.180491.
28. Sathi, F.A.; Paul, S.K.; Ahmed, S.; Alam, M.M.; Nasreen, S.A.; Haque, N.; Islam, A.; Nila, S.S.; Afrin, S.Z.; Aung, M.S.; et al. Prevalence and Antifungal Susceptibility of Clinically Relevant Candida Species, Identification of *Candida auris* and *Kodamaea ohmeri* in Bangladesh. *Trop. Med. Infect. Dis.* **2022**, *7*, 211. https://doi.org/10.3390/tropicalmed7090211.
29. Shuping, L.; Mpembe, R.; Mhlanga, M.; Naicker, S.D.; Maphanga, T.G.; Tsotetsi, E.; Wadula, J.; Velaphi, S.; Nakwa, F.; Chibabhai, V.; et al. Epidemiology of Culture-confirmed Candidemia Among Hospitalized Children in South Africa, 2012–2017. *Pediatr. Infect. Dis. J.* **2021**, *40*, 730–737. https://doi.org/10.1097/INF.0000000000003151.
30. Singh, A.; Mukhopadhyay, S. 585 Changing trends of candida sepsis and its impact on neonates admitted in nicu: A prospective study from a tertiary centre in India. In *British Association of Perinatal Medicine and Neonatal Society*; BMJ Publishing Group Ltd.: London, UK; Royal College of Paediatrics and Child Health: London, UK, 2022; pp. A149.2–A150.
31. Berrio, I.; Caceres, D.H.; Coronell, R.W.; Salcedo, S.; Mora, L.; Marin, A.; Varón, C.; Lockhart, S.R.; Escandón, P.; et al. Bloodstream Infections With *Candida auris* Among Children in Colombia: Clinical Characteristics and Outcomes of 34 Cases. *J. Pediatr. Infect. Dis. Soc.* **2021**, *10*, 151–154. https://doi.org/10.1093/jpids/piaa038.
32. Calvo, B.; Melo, A.S.A.; Perozo-Mena, A.; Hernandez, M.; Francisco, E.C.; Hagen, F.; Meis, J.F.; Colombo, A.L. First report of *Candida auris* in America: Clinical and microbiological aspects of 18 episodes of candidemia. *J. Infect.* **2016**, *73*, 369–374. https://doi.org/10.1016/j.jinf.2016.07.008.
33. Chakrabarti, A.; Sood, P.; Rudramurthy, S.M.; Chen, S.; Jillwin, J.; Iyer, R.; Sharma, A.; Harish, B.N.; Roy, I.; Kindo, A.J.; et al. Characteristics, outcome and risk factors for mortality of paediatric patients with ICU-acquired candidemia in India: A multicentre prospective study. *Mycoses* **2020**, *63*, 1149–1163. https://doi.org/10.1111/myc.13145.
34. Chandramati, J.; Sadanandan, L.; Kumar, A.; Ponthenkandath, S. Neonatal *Candida auris* infection: Management and prevention strategies—A single centre experience. *J. Paediatr. Child Health* **2020**, *56*, 1565–1569. https://doi.org/10.1111/jpc.15019.
35. Chibabhai, V. Incidence of candidemia and prevalence of azole-resistant candidemia at a tertiary South African hospital—A retrospective laboratory analysis 2016–2020. *S. Afr. J. Infect. Dis.* **2022**, *37*, 326. https://doi.org/10.4102/sajid.v37i1.326.
41. Kullberg, B.J.; Arendrup, M.C. Invasive Candidiasis. *N. Engl. J. Med.* **2015**, *373*, 1445–1456. https://doi.org/10.1056/NEJMra1315399.
42. Pfaller, M.A.; Jones, R.N.; Castanheira, M. Regional data analysis of Candida non-albicans strains collected in United States medical sites over a 6-year period, 2006–2011. *Mycoses* **2014**, *57*, 602–611. https://doi.org/10.1111/myc.12206.
43. Guinea, J. Global trends in the distribution of *Candida* species causing candidemia. *Clin. Microbiol. Infect.* **2014**, *20* (Suppl. 6), 5–10. https://doi.org/10.1111/1469-0691.12539.
44. Pfaller, M.A.; Andes, D.R.; Diekema, D.J.; Horn, D.L.; Reboli, A.C.; Rotstein, C.; Franks, B.; Azie, N.E. Epidemiology and outcomes of invasive candidiasis due to non-albicans species of Candida in 2496 patients: Data from the Prospective Antifungal Therapy (PATH) registry 2004–2008. *PLoS ONE* **2014**, *9*, e101510. https://doi.org/10.1371/journal.pone.0101510.
45. Arendrup, M.C.; Dzajic, E.; Jensen, R.H.; Johansen, H.K.; Kjaeldgaard, P.; Knudsen, J.D.; Kristensen, L.; Leitz, C.; Lemming, L.E.; Nielsen, L.; et al. Epidemiological changes with potential implication for antifungal prescription recommendations for fungaemia: Data from a nationwide fungaemia surveillance programme. *Clin. Microbiol. Infect.* **2013**, *19*, E343–53. https://doi.org/10.1111/1469-0691.12212.
79. Morales-López, S.E.; Parra-Giraldo, C.M.; Ceballos-Garzón, A.; Martínez, H.P.; Rodríguez, G.J.; Álvarez-Moreno, C.A.; Rodríguez, J.Y. Invasive Infections with Multidrug-Resistant Yeast *Candida auris*, Colombia. *Emerg. Infect. Dis.* **2017**, *23*, 162–164. https://doi.org/10.3201/eid2301.161497.
